# Thermal and Electronic Transport Properties of the Half-Heusler Phase ScNiSb

**DOI:** 10.3390/ma12101723

**Published:** 2019-05-27

**Authors:** Karol Synoradzki, Kamil Ciesielski, Igor Veremchuk, Horst Borrmann, Przemysław Skokowski, Damian Szymański, Yuri Grin, Dariusz Kaczorowski

**Affiliations:** 1 Institute of Low Temperature and Structure Research, Polish Academy of Sciences, P. O. Box 1410, 50-950 Wrocław, Poland; k.ciesielski@intibs.pl (K.C.); d.szymanski@intibs.pl (D.S.); d.kaczorowski@intibs.pl (D.K.); 2 Max-Planck-Institut für Chemische Physik fester Stoffe, Nöthnitzer Straße 40, 01187 Dresden, Germany; Igor.Veremchuk@cpfs.mpg.de (I.V.); Horst.Borrmann@cpfs.mpg.de (H.B.); Juri.Grin@cpfs.mpg.de (Y.G.); 3 Institute of Molecular Physics, Polish Academy of Sciences, Smoluchowskiego 17, 60-179 Poznań, Poland; przemyslaw.skokowski@ifmpan.poznan.pl

**Keywords:** Heusler alloys, thermoelectric, ScNiSb

## Abstract

Thermoelectric properties of the half-Heusler phase ScNiSb (space group *F*$\bar{4}$3*m*) were studied on a polycrystalline single-phase sample obtained by arc-melting and spark-plasma-sintering techniques. Measurements of the thermopower, electrical resistivity, and thermal conductivity were performed in the wide temperature range 2–950 K. The material appeared as a *p*-type conductor, with a fairly large, positive Seebeck coefficient of about 240 μV K^−1^ near 450 K. Nevertheless, the measured electrical resistivity values were relatively high (83 μΩm at 350 K), resulting in a rather small magnitude of the power factor (less than 1 × 10^−3^ W m^−1^ K^−2^) in the temperature range examined. Furthermore, the thermal conductivity was high, with a local minimum of about 6 W m^−1^ K^−1^ occurring near 600 K. As a result, the dimensionless thermoelectric figure of merit showed a maximum of 0.1 at 810 K. This work suggests that ScNiSb could be a promising base compound for obtaining thermoelectric materials for energy conversion at high temperatures.

## 1. Introduction

Designing efficient thermoelectric generators is considered an important issue in the fight against waste heat, which causes significant financial, natural, and social losses. The major challenge is to find proper materials with seemingly conflicting combinations of their transport properties for the laid-down temperature range of the target application. Half-Heusler (HH) phases with rare-earth (RE) metals have recently been recognized as possible candidates for thermoelectric materials [1,2,3,4,5,6,7,8,9,10,11,12], applicable at high temperatures. These materials show *p*-type behavior. For some of them (e.g., PtYSb), thermoelectric parameter values (e.g., *ZT* = 0.57 at 973 K [8]) are as good as for HH phases without RE [MNiSn, MCoSb (M  =  Ti, Zr, Hf) and XFeSb (X  =  V, Nb, Ta)] before optimization [13,14,15,16,17,18]. In recent years, the on-going intense studies on various RE-based HH phases have been focused on their other remarkable properties, like large magnetocaloric effect, huge magnetoresistance, superconductivity, presence of Dirac states, etc. [19,20,21,22,23,24,25,26].

ScNiSb is a member of the large family of the HH phases, which crystallize with the cubic MgAgAs-type crystal structure (space group *F*$\bar{4}$3*m*, no. 216). The compound was discovered by A. E. Dwight [27]. The first structure refinement from X-ray powder diffraction data confirmed the equiatomic composition of the substance [27,28]. Later, a structure refinement on X-ray single-crystal diffraction data revealed a defect of ca. 14 at % at the nickel position [29]. Based on the results of low-temperature measurements, Oestreich et al. determined for this compound the thermoelectric figure of merit (*ZT*) of an order of 0.024 at 300 K [30,31]. The early calculations performed by Ishida et al. showed that changing site occupations by different chemical constituents should significantly affect the electronic band structure of ScNiSb [32]. The crystallographic disorder (Ni vacancies) was experimentally shown by means of NMR and Mӧssbauer spectroscopy [29]. Nevertheless, the chemical bonding analysis reveals a clear energetic preference for nickel location in the heterocubic site (4b) [33]. Most recent ab initio calculations made by Kocak and Cifti and by Winiarski et al. revealed for ScNiSb the presence of an indirect energy gap [34,35]. The compound was suggested as a good candidate for *p*-type thermoelectric material [35]. Furthermore, the potential of using ScNiSb in tandem with NiMnSb was studied by Attema et al. in the context of spintronics [36,37].

Motivated by the literature data, we decided to investigate high-temperature thermoelectric properties of ScNiSb, which seemed to be not explored before, and low-temperature thermoelectric properties in order to not only compare our results with those already published, but also to search for other physical properties, e.g., superconductivity. This research is a part of our comprehensive studies on thermoelectricity in RE-based HH phases [38,39,40,41,42].

## 2. Materials and Methods 

A polycrystalline sample was synthesized by arc-melting elemental scandium (lumps, 99.9%), nickel (rod, 99.99%), and antimony (lumps, 99.999%) in Ti-gettered argon gas atmosphere. In relation to the intensive evaporation of antimony during the melting, 6% of the nominal mass Sb was added beforehand. The obtained ingots were hand-ground into fine powder. In order to obtain dense bulk samples suitable for thermoelectric property measurements, spark plasma sintering (SPS) was applied (SPS-515 ET, Dr Sinter setup, SDC Fuji, Japan). A consolidation was performed by heating the charge to 950 K at 50 K min^−1^ under uniaxial pressure of 100 MPa and dwelling this temperature for 10 min. The density of the so-casted pellets, determined by the Archimedes method, was over 98% of the theoretical value. 

The prepared material was characterized at room temperature (RT) by X-ray powder diffraction (X′pert Pro PANalytical, Cu*K*α radiation, Almelo, Netherlands). Powder diffraction data were collected using an upgraded Huber G670 type Guinier camera with an imaging plate detector. The large focal circle at 360 mm diameter provides for excellent resolution, in particular with hard X-rays. Due to the quite small unit cell for half-Heusler-type compounds, we took advantage of doubling the number of observable Bragg reflections by using the Mo*K*α doublet of the incident beam. As monochromator, a focusing 1D multilayer optics (AXO Dresden, Dresden, Germany) was used. It provides for high usable intensity, along with excellent suppression of the *K*_β_ component in the direct beam. 

The reflection positions obtained by profile deconvolution were corrected for sample displacement. The structure refinement was done by employing the programs FULLPROF (version 6.30) [43] and WinCSD (version 4.19) [44]. Sample composition was checked by energy-dispersive X-ray (EDX) analysis on a FEI scanning electron microscope (FEI, Hillsboro, OR, USA) equipped with an EDAX Genesis XM4 spectrometer.

The Seebeck coefficient and the electrical resistivity of the sintered samples were measured simultaneously under helium atmosphere in the temperature range 350–950 K using the temperature differential and the four-probe methods, respectively, implemented in commercial equipment Linseis LSR-3 (Linseis Messgeraete GmbH, Selb, Germany) and Ulvac ZEM-3 (ULVAC, Methuen, MA, USA). In these measurements, the temperature difference between the ends of each sample was kept equal to 50 K for the LSR-3 device and 20 K, 30 K, and 40 K for the ZEM-3. The thermal diffusivity was measured in the temperature range from 300 K to 923 K using the laser flash method (NETZSCH LFA-457). 

Low-temperature (2–300 K) measurements of electrical resistivity, specific heat, Seebeck coefficient, and thermal conductivity were carried out on a Physical Property Measurement System (PPMS-9, Quantum Design, San Diego, CA, USA). The electrical resistivity was measured by standard four-point DC technique, where electrical contacts were made from silver wires attached to the sample by silver paste. The heat capacity measurements were carried out using the relaxation method with the two-τ model. For Seebeck and thermal conductivity measurements, gold-plated copper electrodes were attached to the specimen using silver-epoxy paste.

## 3. Results and Discussion

**Figure 1:**
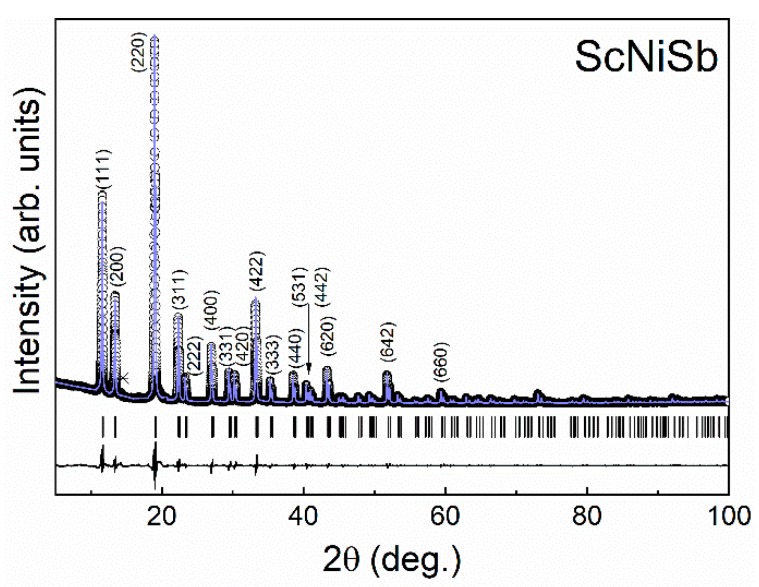
X-ray diffraction pattern of the half-Heusler compound ScNiSb (Mo*K*α radiation). The solid line through the experimental points represents the refinement profile. Black ticks show the angular positions of the reflections of the ScNiSb phase. The asterisk marks a strongest reflection due to the Sc_2_O_3_ phase. The difference pattern is shown as a black solid line on the bottom.

First, crystal structure determination was performed with the X-ray powder diffraction pattern obtained using the Cu*K*α radiation (Bragg–Brentano geometry, 2*θ*_max_ = 90°, 11 reflections available in the measured range). All the Bragg peaks were well indexed with the cubic system (space group *F*4$\bar{3}$*m*), except traces of impurity phase Sc_2_O_3_, spotted around 31.3°. The lattice parameter (*a* = 6.0749(2) Å) obtained is slightly larger than the experimental values reported before in the literature (between 6.0498 Å and 6.0620 Å) [27,28,29,31], yet smaller than the calculated ones [32,34,35]. The differences between experimental values may have resulted from a different level of structural disorder caused, for example, by slightly different stoichiometry (cf. below). The structure refinement was performed first, considering that Sc, Ni, and Sb atoms occupy the 4*b* (½ ½ ½), 4*c* (¼ ¼ ¼), and 4a (0 0 0) sites, respectively, and the occupancy factors were assumed to be equal to unity. Despite the obtained low residuals (*R*_I_ = 0.031, *R*_P_ = 0.043), the atomic displacement parameters reveal non-systematic change with the atomic masses: *B*(Sc) = 1.0(1) Å^2^, *B*(Ni) = 1.3(2) Å^2^, *B*(Sb) = 0.75(7) Å^2^. An attempt to refine the occupation of the Ni site (vacancy on this position was suggested in [29]) was not successful: *R*_I_ = 0.031, *R*_P_ = 0.04; *B*(Sc) = 0.93(1) Å^2^, *B*(Ni) = 1.0(2) Å^2^, *B*(Sb) = 0.78(7) Å^2^; Occ(Ni) = 0.98(1). Another reason for enhanced *B*(Ni) may have been the off-center location of the atoms at this position. Indeed, the Ni could be refined at 16*e* position (*xxx*) with *x* = 0.262(2). This did not change the residuals (*R*_I_ = 0.031, *R*_P_ = 0.043) but allowed a more logical distribution of the atomic displacement parameters to be obtained (*B*(Sc) = 0.98(14) Å^2^, *B*(Ni) = 0.85(15) Å^2^, *B*(Sb) = 0.75(7) Å^2^). Despite the low residuals, the used powder diffraction data did not allow a final decision about the structural details in ScNiSb. To shed more light, high-resolution X-ray powder diffraction data were measured, employing Huber G670 type Guinier camera with double radius and using Mo*K*α radiation (2*θ*_max_ = 100°, 84 reflections available in the measured range). In this experiment, the application of the ideal atomic distribution on the crystallographic sites confirmed the atomic displacement parameters not following the atomic masses (*B*(Sc) = 0.64(3) Å^2^, *B*(Ni) = 0.75(3) Å^2^, *B*(Sb) = 0.61(2) Å; *R*_I_ = 0.027, *R*_P_ = 0.093). An attempt to refine the occupancy of the nickel position did not reveal any vacancies. The stoichiometric composition of the material is in agreement with the lattice parameter, which is clearly larger (*a* = 6.0761(4) Å) than that for the Ni-defect compositions ScNi_0.87_Sb (*a* = 6.0521(6) Å) and Sc Ni_0.85_Sb (*a* = 6.0498(6) Å) [29]. The off-center model with Ni at the 16*e* position (*x* = 0.256(2)) yielded similar residuals (*R*_I_ = 0.024, *R*_P_ = 0.097). Yet the sequence of the atomic displacement parameters (*B*(Sc) = 0.66(3) Å^2^, *B*(Ni) = 0.61(4) Å^2^, *B*(Sb) = 0.59(2) Å) is in much better agreement with the atomic masses of the elements. The final results of the crystal structure refinement of ScNiSb from powder X-ray diffraction (Mo*K*α radiation) data are presented in Figure 1. Further details of the real crystal structure may be revealed using the high-resolution X-ray single-crystal data at the equiatomic composition. Nonetheless, in most possible scenario with off-center Ni atoms, the crystal structure reveals clear deviation from the translation symmetry, which should reduce the lattice thermal conductivity, as was recently shown for the intermetallic clathrates [45].

The experimental sample density obtained by the Archimedes method is only 1.5% smaller than the theoretical value (Table 1). The prepared sample of ScNiSb was hard and brittle, as predicted from theoretical calculations [34].

**Figure 2:**
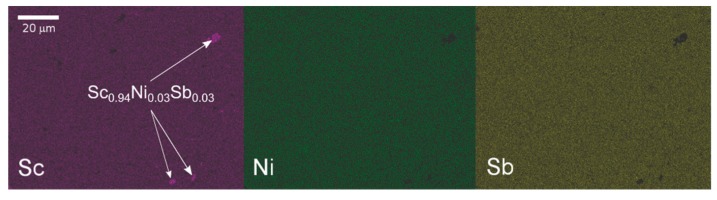
Element mapping of the ScNiSb sample investigated.

The elements distribution on the polished surface of the specimen is presented in Figure 2. Consistent with the PXRD results, the sample appears fairly homogeneous, except for tiny amounts of scandium-rich phase, probably an oxide. The chemical composition derived as an average over three different points examined on the sample surface is in very good agreement with the nominal one (see Table 1). This supports the off-center position of Ni in the crystal structure.

**Figure 3:**
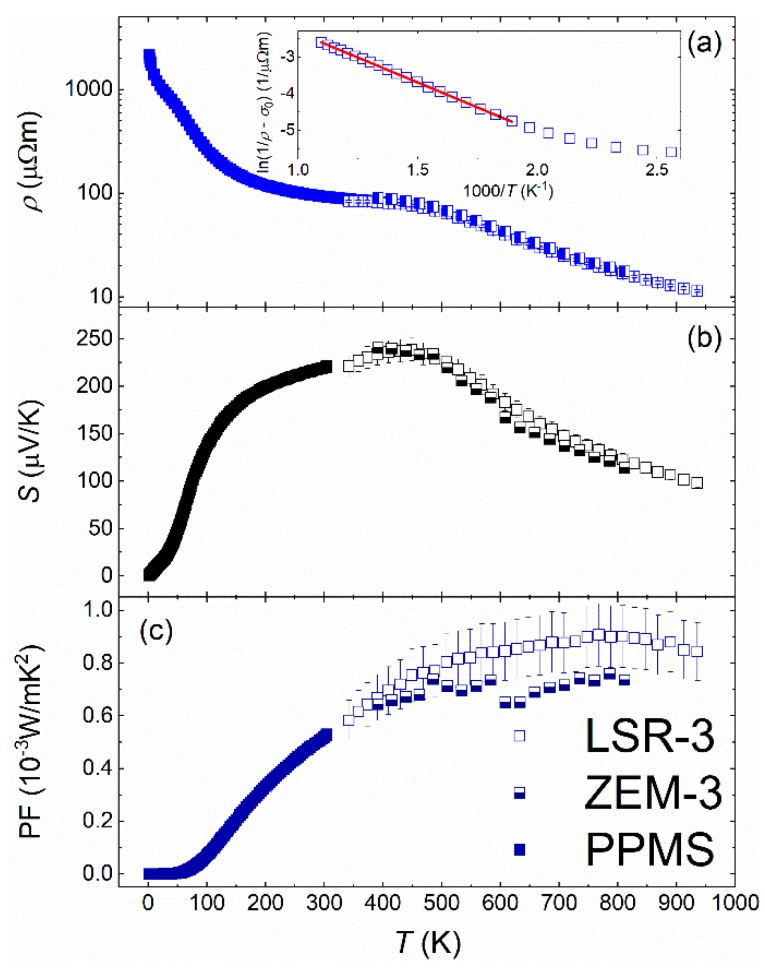
Temperature dependencies of (**a**) electrical resistivity (note semi-logarithmic scale), (**b**) Seebeck coefficient, and (**c**) thermoelectric power factor of ScNiSb. The inset in panel (**a**) shows the high-temperature ln(1/*ρ* −*σ*_0_) data vs. 1000/*T* fitted with the Arrhenius model. Open, half-filled, and filled symbols represent the data collected using LSR-3, ZEM-3, and PPMS device, respectively.

The temperature dependencies of the electrical resistivity (*ρ*) and the Seebeck coefficient (*S*) of ScNiSb, determined in a wide temperature interval, are shown in Figure 3. At elevated temperatures, the experiments carried out on heating and cooling the specimen yielded very similar results, and hence only the data obtained on cooling are shown. Moreover, it should be noted that near 300 K the measurements performed employing different techniques/equipment (LSR-3, ZEM-3, PPMS) converged to almost the same values. Therefore, in the following discussion, the data collected using LSR-3 will be evaluated.

As can be inferred from Figure 3, ScNiSb exhibits semiconducting-like behavior, typical for doped semiconductors, with an ionization (or freeze-out) region from 2 K up to about 150 K, an extrinsic (or saturation) region up to about 500 K, and an intrinsic region at higher temperatures. A broad shoulder observed around 50 K has unclear origin. As shown in the inset to Figure 3a, in the intrinsic region, the resistivity can be well described by a standard Arrhenius model: 1/*ρ* = *σ*_0_ + *σ*exp(−*E*_g_/2*k*_B_*T*), where *σ*_0_ stands for the residual conductivity, and *E*_g_ is the activation energy. The so-derived value of *E*_g_ amounts to 0.47(1) eV, which is much larger than that reported in the literature [31,35,46].

The thermoelectric power of ScNiSb is positive in the entire temperature range studied, because the number of holes in the valence band far exceeds the number of electrons in the conduction band. Therefore, ScNiSb is a *p*-type material. The *S*(*T*) dependence shows a shoulder-like feature at 120 K and a broad maximum (*S*_max_ = 240 μV K^−1^) near *T* = 450 K. This maximum is related to the compensation effect, when the electron concentration starts to overcome the holes concentration. Using the relationship [47] *S*_max_ = *E*_g_/2e*T*_max_ (e stands for elementary charge) one finds *E*_g_ = 0.22 eV, in good agreement with the theoretical data [35,46], however more than twice smaller than that determined from the *ρ* data. It should be noted that the so-obtained value of *E*_g_ may differ from the actual one because of breakdown of the Maxwell–Boltzmann law in a material with narrow energy gap or with strong deviation in carriers mobility [48].

The temperature dependence of the power factor (PF = *S*^2^/*ρ*) calculated from the measured data of ScNiSb is presented in Figure 3c. On increasing temperature, PF starts growing above about 50 K and reaches a maximum of 0.90(4) × 10^−3^ W m K^2^ at 810 K. This value is similar to those determined for other RE-based HH phases [3,5,38,39,40,41,42] and other thermoelectric materials [49].

**Figure 4:**
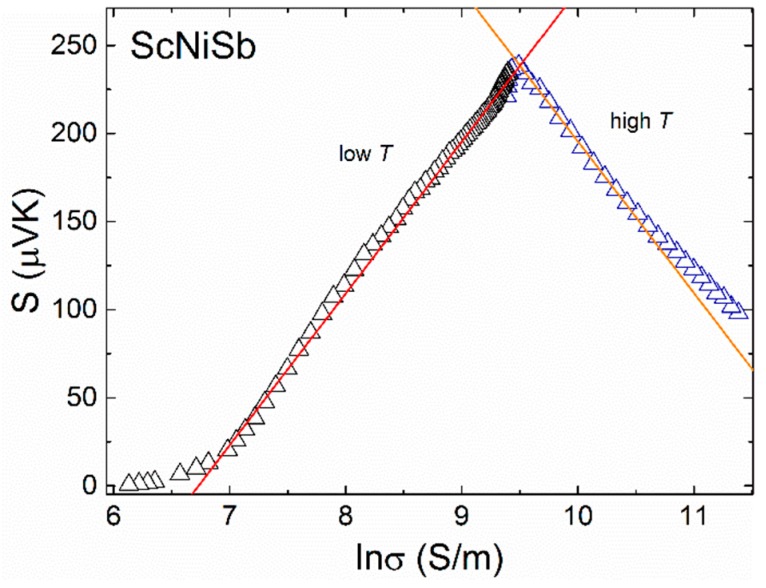
Jonker plot of the electrical conductivity and the Seebeck coefficient data of ScNiSb.

In order to inspect the conduction mechanism in ScNiSb, a Jonker plot was constructed (Figure 4) [50]. The observed linear relationship between the thermopower and logarithm of the electrical conductivity is a characteristic feature of semiconductor in its intrinsic region, with charge carriers scattered mainly on acoustic phonons [51]. At low temperatures, the slope of the straight line is positive, while at high temperatures it is negative. However, in both temperature regions, slope has a constant value of ±86.15 μV K^−1^. The switch in the sign of the Jonker-type correlation occurring near 450 K suggests that the temperature variations of the Seebeck coefficient and the electrical conductivity in ScNiSb are governed mainly by changes in the carrier concentration.

**Figure 5:**
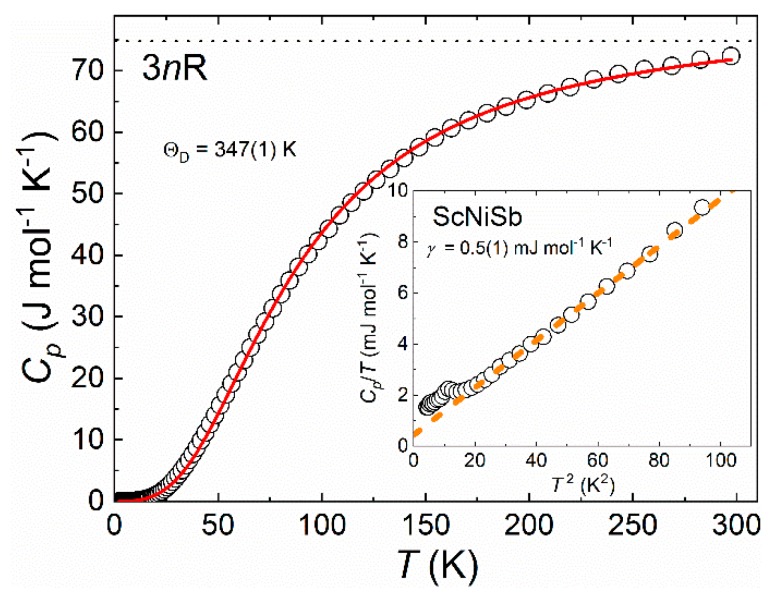
Temperature dependence of the specific heat of ScNiSb. Solid line represents Debye model. Dotted line represents the Dulong–Petit limit (3*n*R). Inset: the low-temperature *C*_p_/*T* vs. *T*^2^ data, dashed line is a linear fit.

The low-temperature (*T* < 300 K) specific heat (*C*_p_) of ScNiSb is featureless, except for little hump near 3 K (see Figure 5). Possibly, the latter anomaly appears because of the impurity phase detected in the PXRD and EDX studies. Generally, the *C*_p_(*T*) of ScNiSb has a shape typical for nonmagnetic compounds and can be analyzed by Debye formula:

(1) $$C_{p}=\;\gamma T+9nR{\left(\frac{T}{\Theta_{D}}\right)}^{3}\int_{0}^{\Theta_{D}/T}\frac{x^{4}e^{x}}{{\left(e^{x}-1\right)}^{2}}dx,$$

where *n* is the number of atoms per formula unit, R is the gas constant, Θ_D_ is the Debye temperature, and *x* = *hν*/*k*_B_*T*. The first term of Equation (1) corresponds to the electronic part, while the second one corresponds to the phonon contribution to the *C*_p_. The electronic specific heat was described using a simple Sommerfeld term *C*_el_ = *γT*; the fit in the range 4.5–7 K yields *γ* = 0.5(1) mJ mol^−1^ K^−2^ (Inset of Figure 5). By fitting the experimental data over the whole temperature range, we derived the Θ_D_ = 354(1) K. Close to room temperature, *C*_p_ is approaching the Dulong–Petit limit of 74.8 J mol^−1^ K^−1^.

The temperature dependence of the thermal conductivity (*κ*) in ScNiSb was calculated with the *T* > 300 K data derived from the measured thermal diffusivity (*D*), using the relationship *κ* = *DC*_p_*d*, where *C*_p_ = 3*n*R represents the specific heat (*n* is a number of atoms in formula unit, and R is the gas constant), while *d* denotes the density of the material. The overall magnitude of *κ* is greater than in the literature results [31]. A small increase of *κ* above about 600 K can be related to heat loses during the measurement or/and some contribution due to bipolar thermal conductivity [52]. At lower temperatures we observed a well-exposed peak at ~50 K, which is related to the interplay between different types of phonon-scattering processes, and suggests high quality of our sample. 

**Figure 6:**
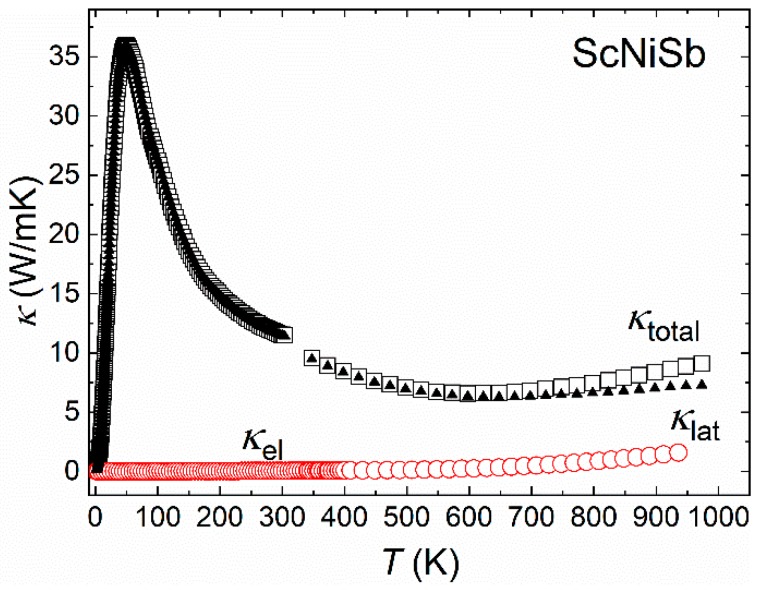
Temperature variation of the thermal conductivity in ScNiSb (squares). Estimates for the electron (*κ*_el_) and lattice contributions (*κ*_lat_) (see the text) are shown by red circles and black triangles, respectively.

Assuming the validity of the Wiedemann–Franz law, *κ*_el_ = *LσT*, where L is the Lorenz number, one can calculate the electronic contribution (*κ*_el_) to total *κ*. Shown in Figure 6 is the estimate of *κ*_el_ in ScNiSb, derived with *L* = 1.5 + exp(−|*S*|/116), as given in Ref. [53]. The so-obtained *κ*_el_ is fairly small and slightly increasing with increasing temperature. This result implies that the thermal conductivity in ScNiSb is dominated in the whole temperature range studied by the lattice contribution (*κ*_lat_). Remarkably, the magnitude of *κ*_lat_ is much larger than the minimum thermal conductivity calculated using the Cahill model [54]. This finding opens a prospective of significant reducing *κ*_lat_ by proper alloying and forming composite materials based on ScNiSb. The values between 5 and 10 W m^−1^ K^−1^ above RT are typical for the HH phases [55,56]. The deviations from the translational symmetry found during the crystal structure determination do not reduce markedly the thermal conductivity, as was found recently in intermetallic clathrates [45], raising once more the question of the real atomic structure of the HH phases, as was already discussed for example TiGePt [57,58]. On the other hand, the good thermal conductivity may be understood from the point of view of chemical bonding. The latter characterized by the presence of three-center Sc–Ni–Sb and two-center Sc–Ni interactions. Due to the predominant role of the first type, the bonding may be considered as pseudo homogeneous, i.e., all interactions are same or similar. The regular distribution of similar bonds in the crystal structure of the chemical bonding is described as isotrop. This characteristic of bonding should not influence the thermal conductivity [59].

**Figure 7:**
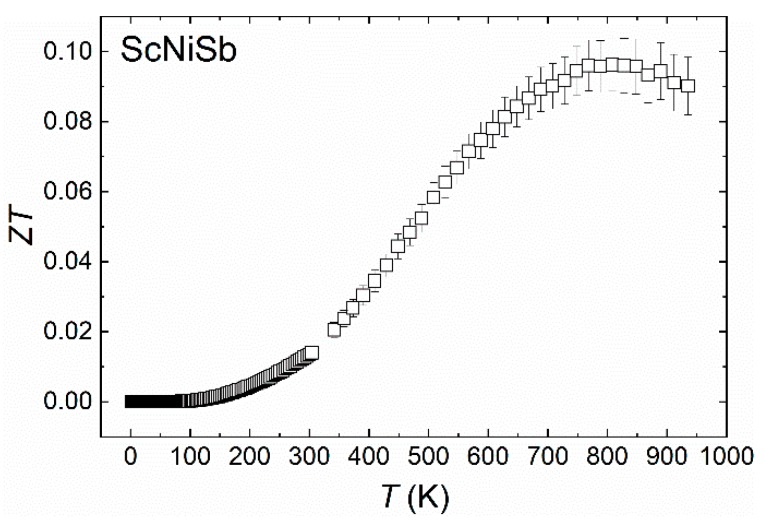
Thermoelectric figure of merit of ScNiSb as a function of temperature.

The experimental data collected for ScNiSb allowed us to calculate the thermoelectric figure of merit (*ZT* = *S*^2^*T*/*ρκ*), and the result is shown in Figure 7. With increasing temperature, *ZT* increased, reaching the maximum *ZT* = 0.10 at 810 K. This value is smaller than *ZT* reported for well-established *p*-type thermoelectrics [60], however it is similar to those found for other RE-based HH phases [1,3,51]. At room temperature *ZT* = 0.01, which is almost two times smaller than the value reported before for an arc-melted sample [31], yet four times larger when compared with *ZT* of our sample, prepared by high-pressure high-temperature (HPHT) sintering [42]. The main reason for the reduced *ZT* values is very low electrical conductivity, opening a way for enhancing the thermoelectric figure of merit by appropriate substitutions.

## 4. Conclusions

As an extension of the literature data for *T* < 400 K, the thermoelectric properties of the HH antimonide ScNiSb were determined from 2 K up to 950 K. Although this material has a high positive value of the Seebeck coefficient (up to 240 μV K^−1^ at 450 K), its thermoelectric properties are moderate. Because of a high electrical resistivity (~100 μΩm around RT) and a relatively high value of thermal conductivity (>6 W m^−1^ K^−1^), the maximum PF and *ZT* values of 0.91(4) × 10^−3^ W m^−1^ K^−2^ and 0.1 at 810 K were established, respectively. 

The results obtained for ScNiSb are similar to the data reported for many other RE-bearing HH phases and for pure RE-free HH phases. It appears plausible that proper modification of this material (nanostructurization, substitution, composite formation, etc.) may lead to significant improvement of its thermoelectric performance.

## Figures and Tables

**Table 1:** Microstructural parameters determined for the studied sample of ScNiSb.

Nominal Composition	Estimated Composition	*a* (Å)	*V* (Å^3^)	Theoretical Density (g/cm^3^)	Measured Density (g/cm^3^)
33.3:33.3:33.3	33.7(4):32.9(2):33.4(2)	6.0761(4)	224.32(5)	6.674(2)	6.58(1)
